# Supplementary material for: Raman Study of Block Copolymers of Methyl Ethylene Phosphate with Caprolactone and L-lactide
Source: Polymers (Basel). 2022 Dec 8;14(24):5367. doi: 10.3390/polym14245367 (PMC9782745; doi:10.3390/polym14245367)
Supplement: Supplementary file 1 [file polymers-14-05367-s001.zip › polymers-2030484-supplementary.pdf]

Supplementary Materials

Raman Study of Block Copolymers of Methyl Ethylene Phosphate with Caprolactone and L-lactide

S.O. Liubimovskii, V.S. Novikov, A.V. Shlyakhtin, V.V. Kuzmin, M.M. Godyaeva, S.V. Gudkov, E.A. Sagitova, L.Yu. Ustynyuk, G.Yu. Nikolaeva

S1. Synthesis

Synthesis of $mPEG_{2000}$—b—PCL copolymers

A preheated glass ampoule of a required volume was equipped with a magnetic stir bar, 1.00 g $mPEG_{2000}$ (1 eq.) was placed into the ampule and then the ampule was filled with dry argon and closed with a septum. Then, 5.0 ml CH_2Cl_2 was added, and after mPEG dissolution, 0.5 ml 1M stock solution in THF of M1 catalyst (5×10^{-4} mol, 1 eq.) was added at 5 °C. After 4 h of stirring at room temperature, 47 ml of dry CH_2Cl_2 was added for **PCL5** sample (66 ml for **PCL6** sample, 20 ml for **PCL4** sample, in order to provide approx. 2M of CL concentration), and then the required amount of CL (0.12 mol (240 eq.) for **PCL5**, 0.17 mol (340 eq.) for **PCL6**, 0.05 mol (100 eq.) for **PCL4**) was added. After 18 h of stirring at room temperature, the reaction mixture was neutralized with an excess of acetic acid, diluted with CH_2Cl_2, and was precipitated twice in diethyl ether and subsequently dried in vacuo. The yield was 11.7 g (80 %) for **PCL5**, 15.6 g (76 %) for **PCL6**, 4.7 g (70 %) for **PCL4**. The ^{1}H NMR spectra of the copolymers are presented in Figure S1.

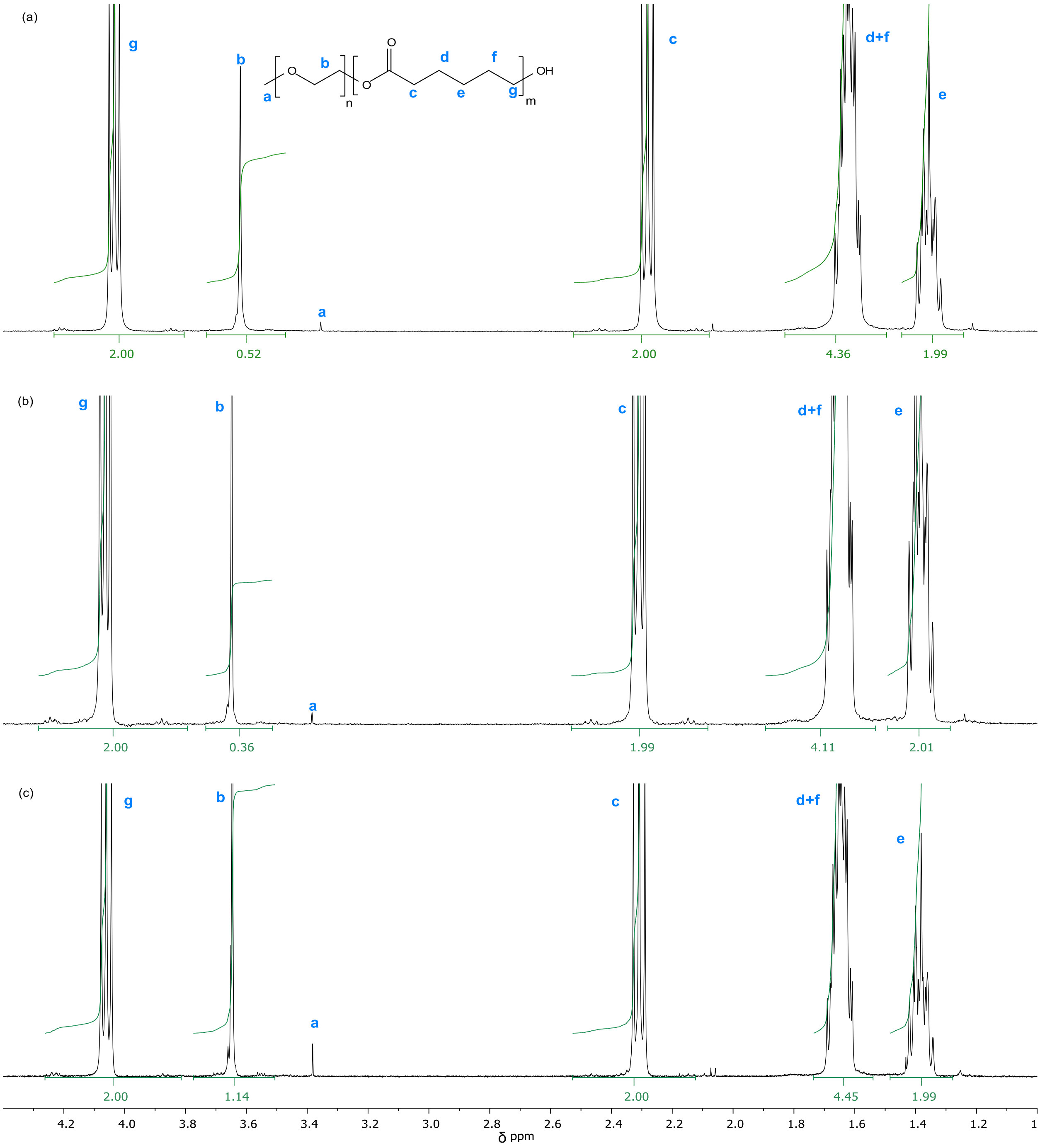

Figure S1: ^{1}H NMR spectra of mPEG$_{2000}$—b—PCL copolymers **PCL5** (a), **PCL6** (b), **PCL4** (c). The signals of the protons of copolymers fragments are marked as letters.

Synthesis of mPEG$_{5000}$—b—PCL copolymer

A preheated 30 ml glass ampoule was equipped with a magnetic stir bar, 1.00 g mPEG$_{5000}$ (1 eq) was placed into the ampule and then the ampule was filled with dry argon and closed with a septum. Then, 5.0 ml CH_2Cl_2 was added, and after mPEG dissolution, 0.2 ml of 1M stock solution in THF of M1 catalyst (2×10^{-4} mol, 1 eq) was added. After 4 h of stirring, 8 ml of dry CH_2Cl_2 and 0.02 mol (100 eq) of CL were added consequently. After 18 h of stirring at room temperature, the reaction mixture was neutralized with an excess of acetic acid, diluted with CH_2Cl_2, and was precipitated twice in diethyl ether and subsequently dried in vacuo. The yield was 1.9 g (58 %). The ^{1}H NMR spectra of copolymer **PCL1** are presented in Figure S2.

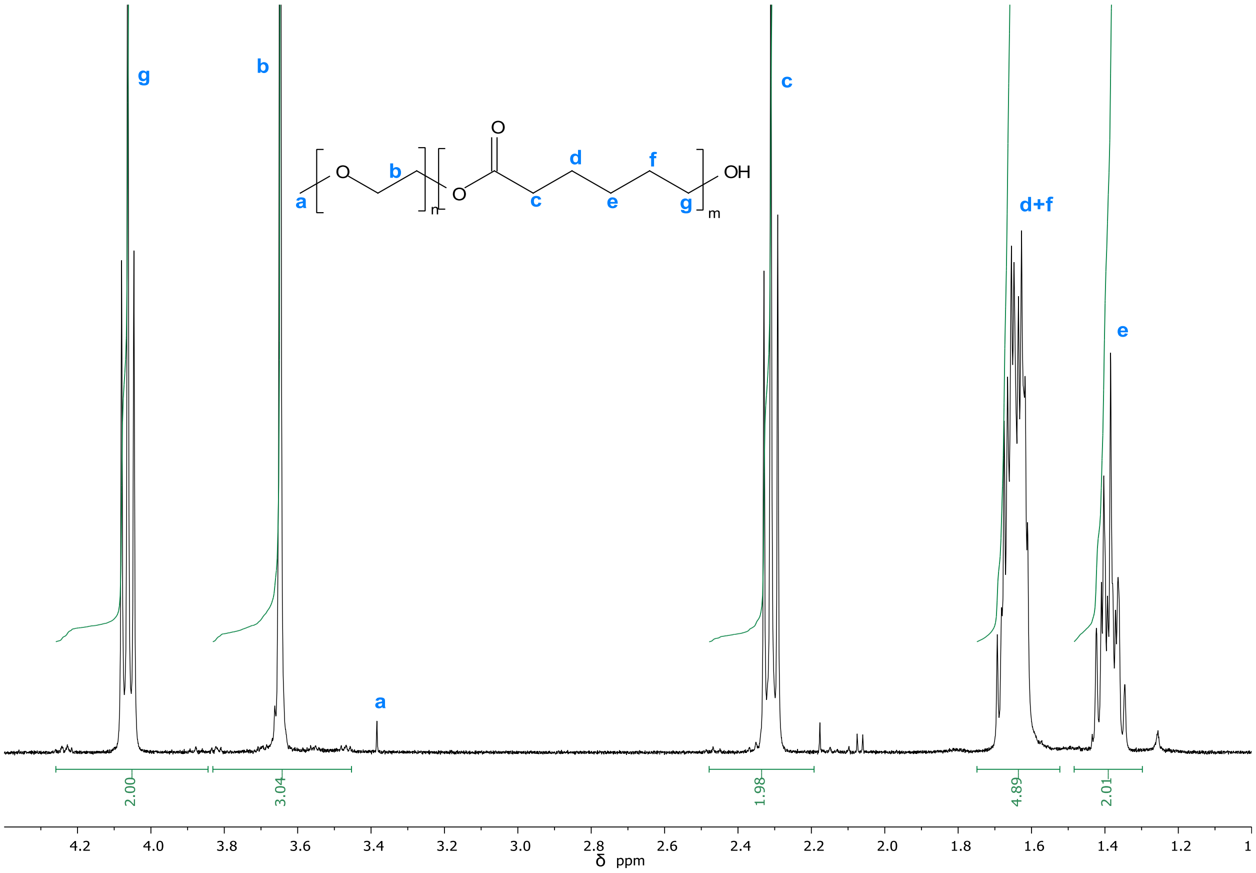

Figure S2: ^{1}H NMR spectrum of mPEG$_{5000}$—b—PCL copolymer (**PCL1**). The signals of the protons of copolymer fragments are marked as letters.

Synthesis of mPEG$_{550}$—b—PCL—b—PMeOEP copolymer 23% PMeOEP

A preheated 100 ml glass ampoule was equipped with a magnetic stir bar, 0.50 g mPEG$_{550}$ (9.1×10^{-4} mol, 1 eq.) was placed into the ampule and then the ampule was filled with dry argon and closed with a septum. Then, 5.0 ml CH_2Cl_2 was added, then 0.9 ml of 1M stock solution in THF of M1 catalyst (9.1×10^{-4} mol, 1 eq.). After 4 h of stirring, 29 ml of dry CH_2Cl_2 was added (to provide approx. 2M of CL concentration), and then 0.091 mol (10.38 g, 10.1 ml, 100 eq.) of CL were added. After 4 h of stirring, MeOEP (0.091 mol, 12.55 g, 9.09 ml, 100 eq.) was added to the reaction mixture. After 4 h of additional stirring, the reaction mixture was neutralized with an excess of acetic acid, polymer solution was precipitated twice in diethyl ether and subsequently dried in vacuo. The yield was 16.3 g (69 %). The ^{1}H and ^{31}P NMR spectra of the copolymer are presented in Figure S3a.

Synthesis of mPEG$_{550}$—b—PCL—b—PMeOEP copolymer 86% PMeOEP

A preheated 50 ml glass ampoule was equipped with a magnetic stir bar, 0.50 g mPEG$_{550}$ (9.1×10^{-4} mol, 1 eq.) was placed into the ampule and then the ampule was filled with dry argon and closed with a septum. Then, 5.0 ml CH_2Cl_2 was added, then 0.9 ml of 1M stock solution in THF of M1 catalyst (9.1×10^{-4} mol, 1 eq.). After 4 h of stirring, 0.0091 mol (1.04 g, 1.01 ml, 10 eq.) of CL were added. After 4 h of stirring, MeOEP (0.027 mol, 3.73 g, 2.70 ml, 30 eq.) was added to the reaction mixture. After 4 h of additional stirring, the reaction mixture was neutralized with an excess of acetic acid, polymer solution was precipitated twice in diethyl ether and subsequently dried in vacuo. The yield was 2.5 g (59 %). The ^{1}H and ^{31}P NMR spectra of the copolymer are presented in Figure S3b.

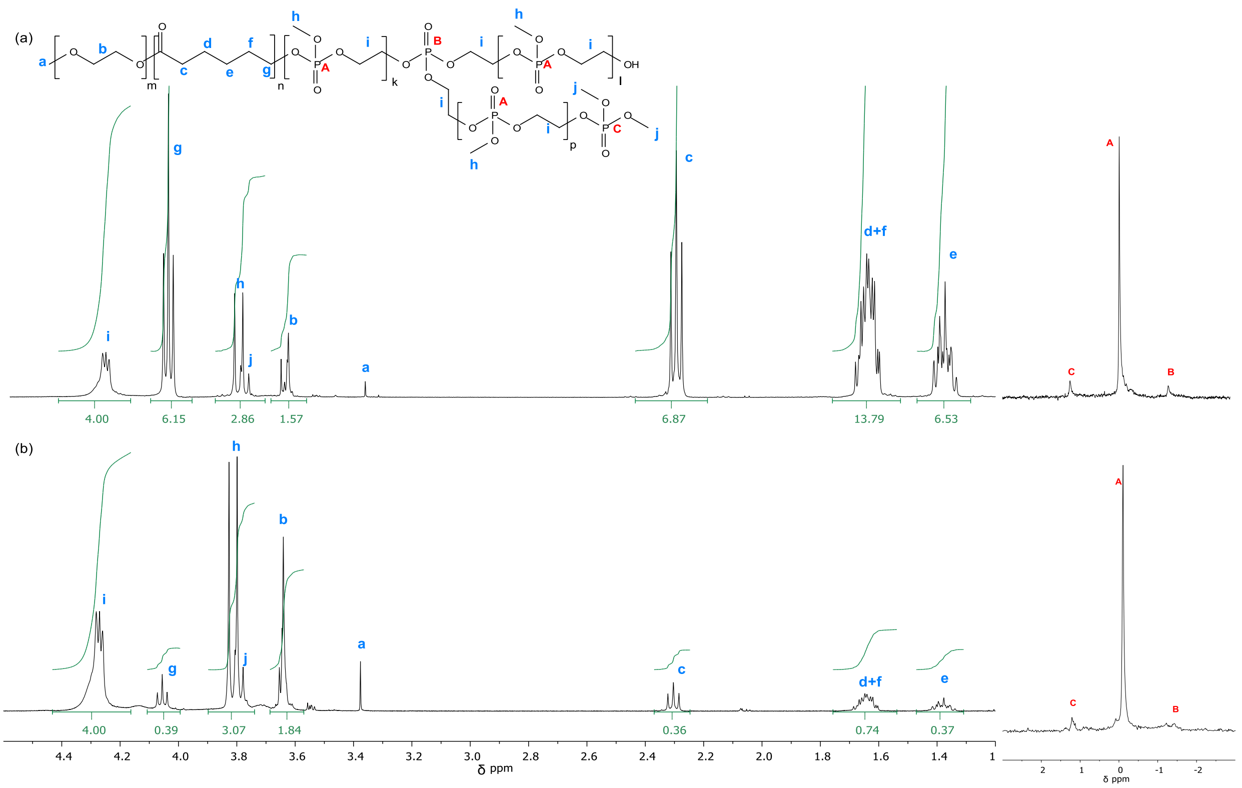

Figure S3: ^{1}H, ^{31}P NMR spectra of mPEG$_{5000}$—b—PCL—b—PMeOEP copolymers **23% PMeOEP** (a), **86% PMeOEP** (b). The signals of the protons and phosphorus atoms of copolymers fragments are marked as letters.

Synthesis of polycaprolactone PCL3

CL (1.31 ml, 1.35 g, 11.8 mmol, 100 eq.) was placed into the preheated 30 ml glass ampule, equipped with a magnetic stir bar and septum, THF (3.6 ml) was added. The reaction mixture was cooled to 5 °C, and a solution of 50 mg [(BHT)Mg(μ-Obn)(THF)]$_2$ (cat M2, 1.18×10^{-4} mol Mg, 1 eq.) in THF (1.0 ml) was added (resulting concentration of CL was 2M). After 8 h of stirring at room temperature, the reaction mixture was neutralized with a 5-fold excess of acetic acid, diluted with dichloromethane, precipitated in diethyl ether twice and dried in vacuo. The yield was 1.19 g (88 %). The ^{1}H NMR spectrum is presented in Figure S4.

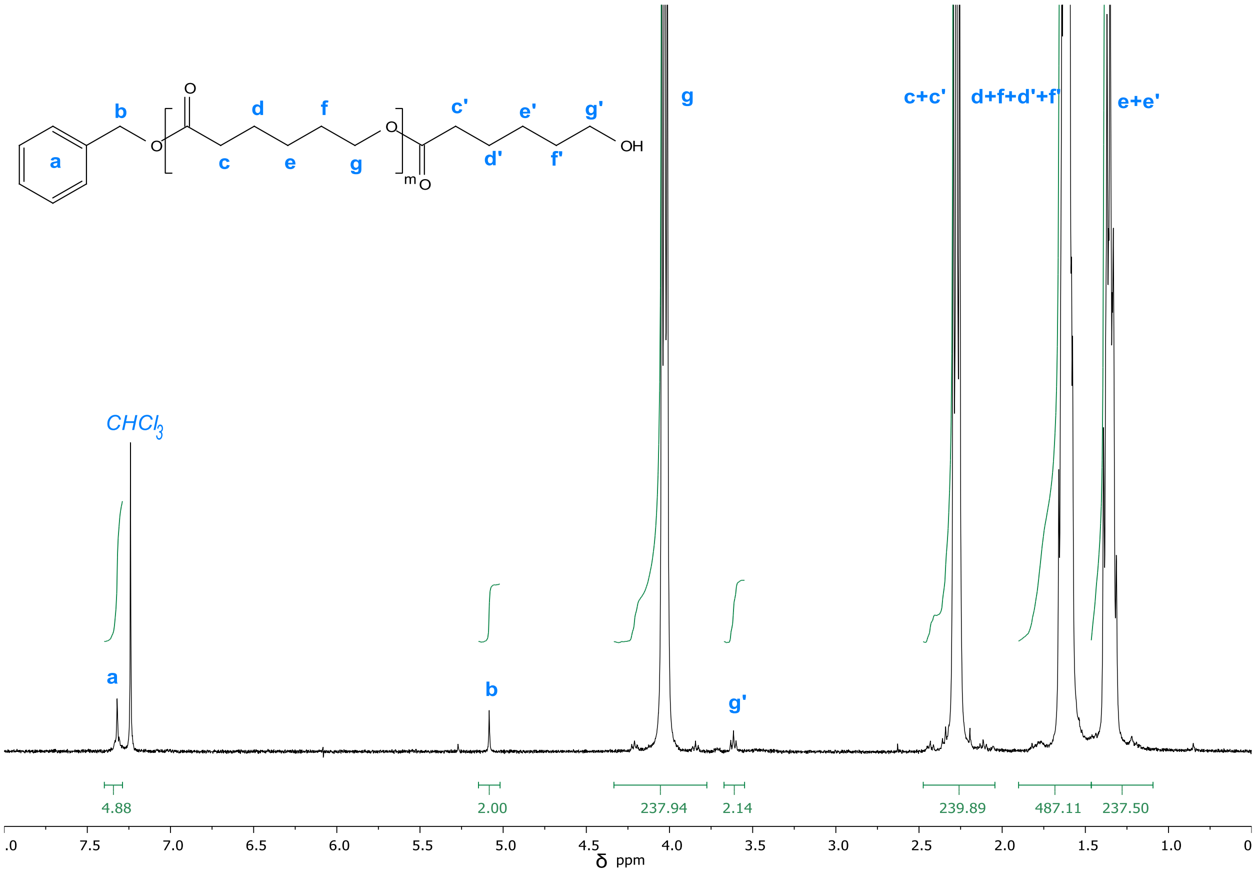

Figure S4: ^{1}H NMR spectrum of BnO—PCL polymer **PCL3**.

Synthesis of poly(methyl ethylene phosphate)s

A preheated 30 ml glass ampoule was equipped with a magnetic stir bar, 1.63 g of MeOEP (1.18 ml, 11.8 mmol, 100 eq.) was placed into the ampule and then the ampule was filled with dry argon and closed with a septum. Then, 4.2 ml of CH_2Cl_2 was added, then the reaction mixture was cooled to 5 °C, and a solution of 50 mg of $[(BHT)Mg(\mu\text{-}Obn)(THF)]_2$ in 0.5 ml THF was added (resulting concentration of MeOEP was 2M). After 1 h of stirring, the reaction mixture was neutralized with an excess of acetic acid, the polymer solution was precipitated twice in diethyl ether and subsequently dried in vacuo. The yield was 1.17 g (72 %) for **PMeOEP2**, 1.26 g (77 %) for **PMeOEP1**.The ^{1}H and ^{31}P NMR spectra of the copolymers are presented in Figure S5.

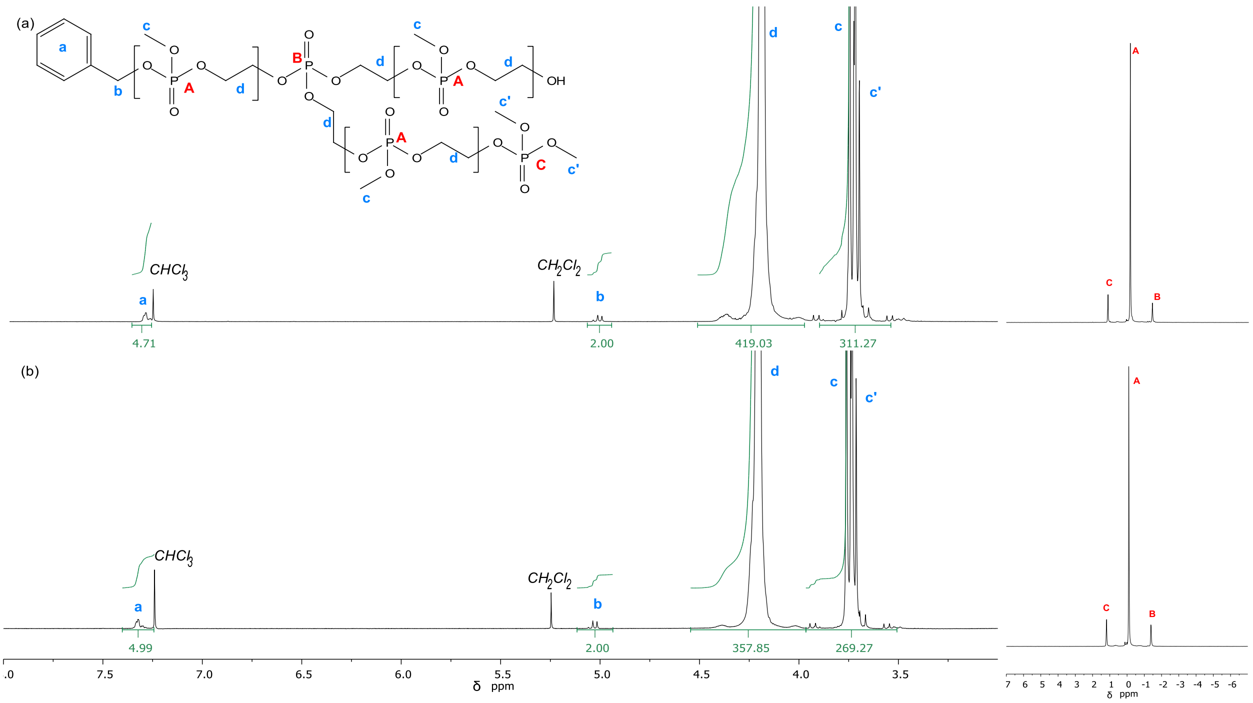

Figure S5: ^{1}H, ^{31}P NMR spectra of BnO—PMeOEP polymers **PMeOEP2** (a), **PMeOEP1** (b).

Synthesis of BnO—PCL—b—PMeOEP copolymer 5% PMeOEP

CL (3.27 ml, 3.37 g, 29.5 mmol, 250 eq.) was placed into the preheated 50 ml glass ampule, equipped with a magnetic stir bar and septum, the ampule was filled with dry argon, CH_2Cl_2 (10.5 ml) was added. The ampule was cooled to 5 °C, and a solution of 50 mg of [(BHT)Mg(μ-Obn)(THF)]$_2$ (1.18×10^{-4} mol, 1 eq.) in 1.0 ml THF was added (resulting concentration of CL was 2M).

After 4 h of stirring at room temperature, the ampule was cooled to 5 °C, and 0.32 g MeOEP (2.36 mmol, 0.24 ml, 20 eq.) was added. After 1 h of stirring at 5 °C, the reaction mixture was neutralized with an excess of acetic acid, polymer solution was diluted with CH_2Cl_2, precipitated twice in diethyl ether and subsequently dried in vacuo. The yield was 3.3 g (89 %). The ^{1}H NMR spectra of the copolymer are presented in Figure S6a.

Synthesis of BnO—PCL—b—MeOEP copolymers (15% PMeOEP, 16% PMeOEP)

CL (3.27 ml, 3.37 g, 29.5 mmol, 250 eq.) was placed into the preheated 50 ml glass ampule, equipped with a magnetic stir bar and septum, the ampule was filled with dry argon, CH_2Cl_2 (10.5 ml) was added. The ampule was cooled to 5 °C, and a solution of 50 mg of [(BHT)Mg(μ-Obn)(THF)]$_2$ (1.18×10^{-4} mol, 1 eq.) in 1.0 ml THF was added (resulting concentration of CL was 2M).

After 4 h of stirring at room temperature, the ampule was cooled to 5 °C, and 0.98 g MeOEP (7.08 mmol, 0.71 ml, 60 eq.) was added. After 6 h of stirring at 5 °C, the reaction mixture was neutralized with an excess of acetic acid, polymer solution was diluted with CH_2Cl_2, precipitated twice in diethyl ether and subsequently dried in vacuo. The yield was 3.2 g (73 %) for **15% PMeOEP**, 3.4 g (78 %) for **16% PMeOEP** copolymer. The ^{1}H NMR spectra of the copolymers are presented in Figures S6b and S6c.

Synthesis of BnO—PCL—b—PMeOEP copolymer 6% PMeOEP

CL (2.62 ml, 2.70 g, 23.6 mmol, 200 eq.) was placed into the preheated 50 ml glass ampule, equipped with a magnetic stir bar and septum, the ampule was filled with dry argon, CH_2Cl_2 (8.5 ml) was added. The ampule was cooled to 5 °C, and a solution of 50 mg of $[(BHT)Mg(\mu\text{-}OBn)(THF)]_2$ (1.18×10^{-4} mol, 1 eq.) in 1.0 ml THF was added (resulting concentration of CL was 2M).

After 4 h of stirring at room temperature, the ampule was cooled to 5 °C, and 0.16 g MeOEP (1.18 mmol, 0.12 ml, 10 eq.) was added. After 6 h of stirring at 5 °C, the reaction mixture was neutralized with an excess of acetic acid, polymer solution was diluted with CH_2Cl_2, precipitated twice in diethyl ether and dried in vacuo. The yield was 2.5 g (87 %). The ^1H NMR spectra of the copolymer are presented in Figure S6d.

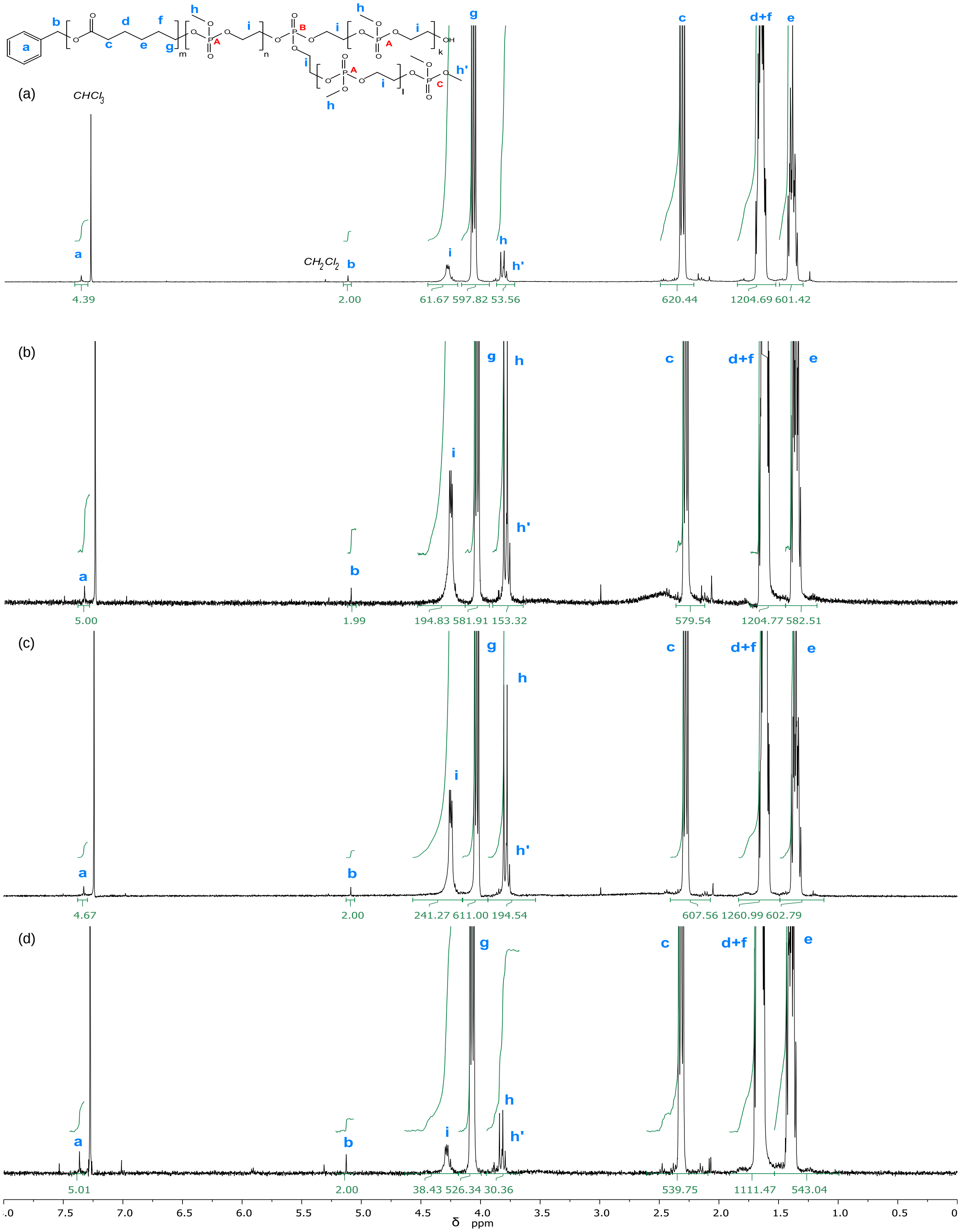

Figure S6: ^{1}H NMR spectra of BnO—PCL—b—PMeOEP copolymers **5% PMeOEP** (a), **15% PMeOEP** (b), **16% PMeOEP** (c), **6% PMeOEP** (d).

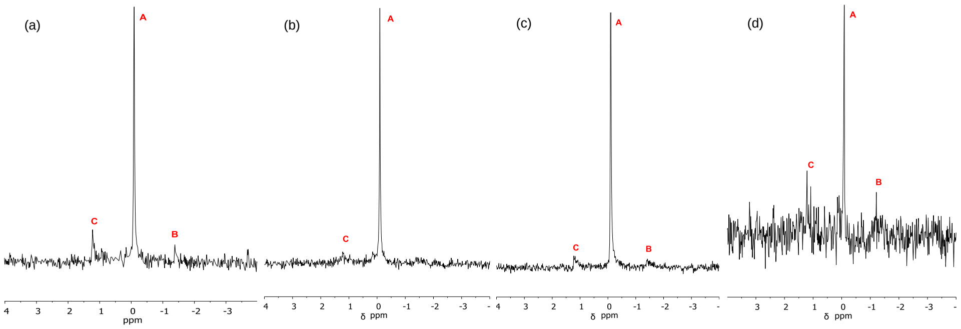

Figure S7: ^{31}P NMR spectra of BnO—PCL—b—PMeOEP copolymers **5% PMeOEP** (a), **15% PMeOEP** (b), **16% PMeOEP** (c), **6% PMeOEP** (d), see reference on the Figure S6a.

Synthesis of poly(L-lactide) PLA1

A preheated 30 ml glass ampoule, equipped with a magnetic stir bar and septum, was filled with dry argon. Afterward, 852 mg of LA (5.9×10^{-3} mol, 50 eq.) was placed into the ampule and then the ampule was purged with dry argon and closed with a septum. Next, 1.6 ml of CH_2Cl_2 was added, and after LA dissolution, a solution of 50 mg of $[(BHT)Mg(\mu\text{-}OBn)(THF)]_2$ in 0.5 ml THF (1.18×10^{-4} mol, 1 eq.) was added (resulting concentration of LA was approx. 2M). After 1 h of stirring, the reaction mixture was neutralized with an excess of acetic acid, the polymer solution was precipitated twice in methanol and dried in vacuo. The yield was 0.58 g (68 %). The ^{1}H NMR spectrum of polymer **PLA1** is presented in Figure S8a.

Synthesis of poly(L-lactides) PLA3, PLA2

A preheated 30 ml glass ampoule, equipped with a magnetic stir bar and septum, was filled with dry argon. Afterward, 1.70 g of LA (1.2×10^{-2} mol, 100 eq.) was placed into the ampule and then the ampule was purged with dry argon and closed with a septum. Next, 3.8 ml of CH_2Cl_2 was added, and after LA dissolution, a solution of 50 mg of $[(BHT)Mg(\mu\text{-}OBn)(THF)]_2$ in 0.5 ml THF (1.18×10^{-4} mol, 1 eq.) was added (resulting concentration of LA was approx. 2M). After 4 h of stirring, the reaction mixture was neutralized with an excess of acetic acid, polymer solution was diluted with CH_2Cl_2 and precipitated twice in methanol and dried in vacuo. The yield was 1.38 g (81 %) for **PLA3**, 1.44 g (85 %) for **PLA2** sample. The ^{1}H NMR spectra of polymers are presented in Figures S8b and S8c.

Synthesis of poly(L-lactide) PLA5

A preheated 50 ml glass ampoule, equipped with a magnetic stir bar and septum, was filled with dry argon. Afterward, 6.80 g of LA (4.7×10^{-2} mol, 400 eq.) was placed into the ampule and then the ampule was purged with dry argon and closed with a septum. Next, 16 ml of CH_2Cl_2 was added, and after LA dissolution, a solution of 50 mg of $[(BHT)Mg(\mu\text{-}OBn)(THF)]_2$ in 1 ml THF (1.18×10^{-4} mol, 1 eq.) was added (resulting concentration of LA was approx. 2M). After 8 h of stirring, the reaction mixture was neutralized with an excess of acetic acid, polymer solution was diluted with CH_2Cl_2, precipitated twice in methanol and dried in vacuo. The yield was 6.47 g (95 %). The ^{1}H NMR spectrum of polymer **PLA5** is presented in Figure S8d.

Synthesis of poly(L-lactide) PLA4

A preheated 100 ml glass ampoule, equipped with a magnetic stir bar and septum, was filled with dry argon. Afterward, 8.50 g of LA (5.9×10^{-2} mol, 500 eq.) was placed into the ampule and then the ampule was purged with dry argon and closed with a septum. Next, 20 ml of CH_2Cl_2 was added, and after LA dissolution, a solution of 50 mg of $[(BHT)Mg(\mu\text{-}OBn)(THF)]_2$ in 1 ml THF (1.18×10^{-4} mol, 1 eq.) was added (resulting concentration of LA was approx. 2M). After 8 h of stirring, the reaction mixture was neutralized with an excess of acetic acid, the polymer solution was diluted with CH_2Cl_2, precipitated twice in methanol and dried in vacuo. The yield was 8.04 g (94 %). The ^1H NMR spectrum of polymer **PLA4** is presented in Figure S8e.

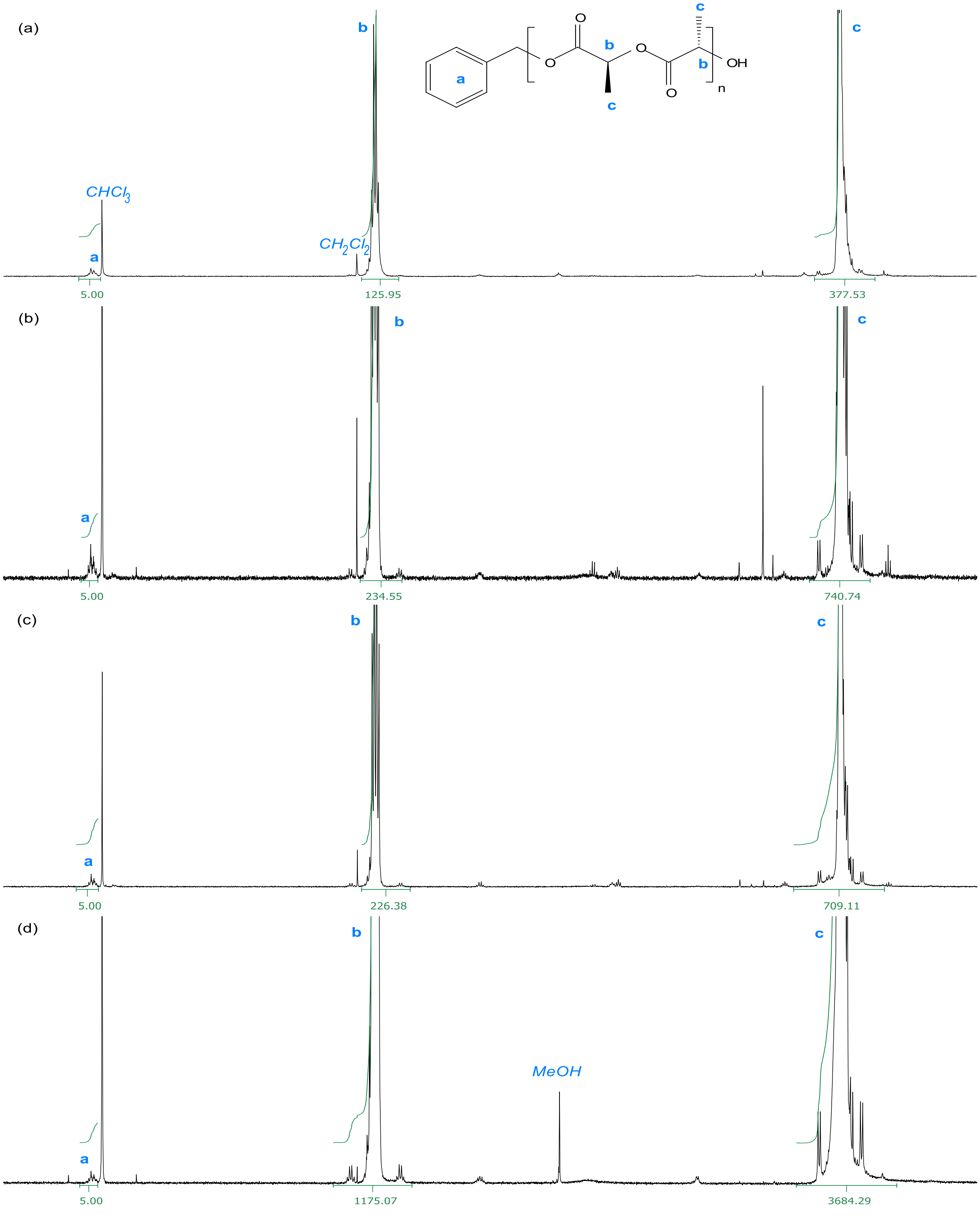

(a)
(b)
(c)
(d)
a
b
c
OH
n
O
CHCl3
CH2Cl2
MeOH
5.00
125.95
377.53
5.00
234.55
740.74
5.00
226.38
709.11
5.00
1175.07
3684.29

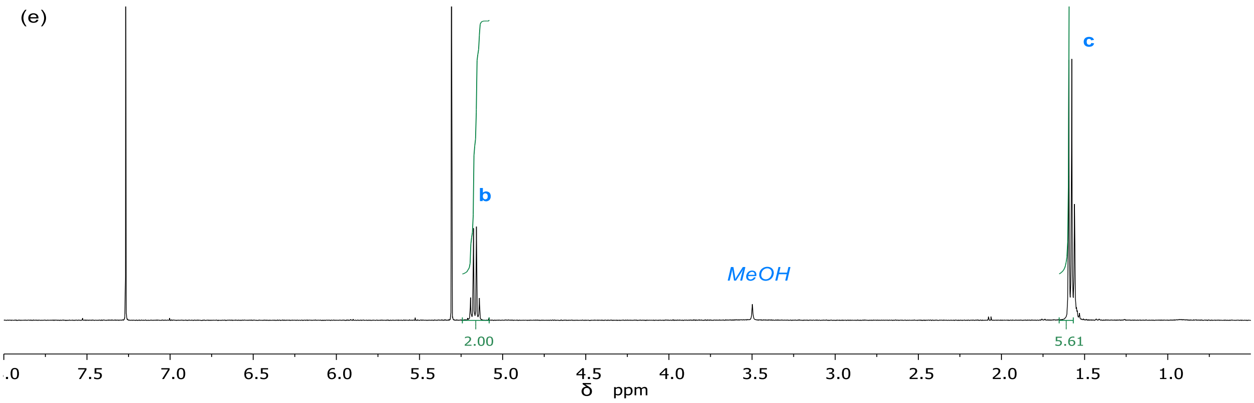

Figure S8: ^{1}H NMR spectra of BnO—PLA polymers **PLA1** (a), **PLA3** (b), **PLA2** (c), **PLA5** (d), **PLA4** (e).

Synthesis of mPEG$_{5000}$—b—PLA copolymer PLA6

A preheated 10 ml glass ampoule was equipped with a magnetic stir bar, 1.00 g mPEG$_{5000}$ (1 eq., 0.2 mmol) was placed into the ampule and then the ampule was filled with dry argon and closed with a septum. Then, 5.0 ml CH_2Cl_2 was added, and after mPEG dissolution, 0.2 ml of 1M stock solution in THF of **M2** catalyst (2×10^{-4} mol, 1 eq.) was added. The mixture was stirred for 4 hours.

Next, 11.5 g of LA (400 eq., 80 mmol) was placed in a 100 ml preheated dry glass ampoule with a magnetic stir bar. The ampoule was filled with dry argon and closed with a septum. Dry dichloromethane was added to LA up to 50 ml to provide approx. 1.5 M monomer concentration. After LA dissolution, the solution of the catalyst was added via syringe at room temperature and the reaction mixture was stirred for an additional 4 hours.

After that, the reaction mixture was neutralized with an excess of acetic acid, diluted with CH_2Cl_2, and it was precipitated twice in methanol and subsequently dried in vacuo. The yield was 9.3 g (74 %). The ^{1}H NMR spectra of the copolymer are presented in Figure S9.

Synthesis of BnO—b—PMeOEP—b—PLA copolymer 31% PMeOEP

First, 1.70 g of LA (100 eq., 11.8 mmol) was placed in a 30 ml preheated dry glass ampoule with a magnetic stir bar. The ampoule was filled with dry argon and closed with a septum. Dry dichloromethane was added to LA up to 8 ml to provide approx. 1.5 M monomer concentration.

MeOEP (0.59 ml, 814 mg, 5.9 mmol, 50 eq.) was placed into the preheated 10 ml glass ampule, equipped with a magnetic stir bar and septum, the ampule was filled with dry argon, 1.9 ml of dry dichloromethane was added via syringe. The ampule was cooled to 5 °C, and a solution of 50 mg of $[(BHT)Mg(\mu\text{-}OBn)(THF)]_2$ (1.18×10^{-4} mol, 1 eq.) in 0.5 ml THF was added (resulting concentration of MeOEP was 2M). After 5 min of stirring at 5 °C, the reaction mixture was added to LA solution at room temperature via syringe. After 4 h of stirring, the reaction mixture was neutralized with an excess of acetic acid, the polymer solution was diluted with CH_2Cl_2, precipitated twice in diethyl ether and dried in vacuo. The yield was 2.0 g (80 %). The ^{1}H NMR spectrum of the copolymer is presented in Figure S10.

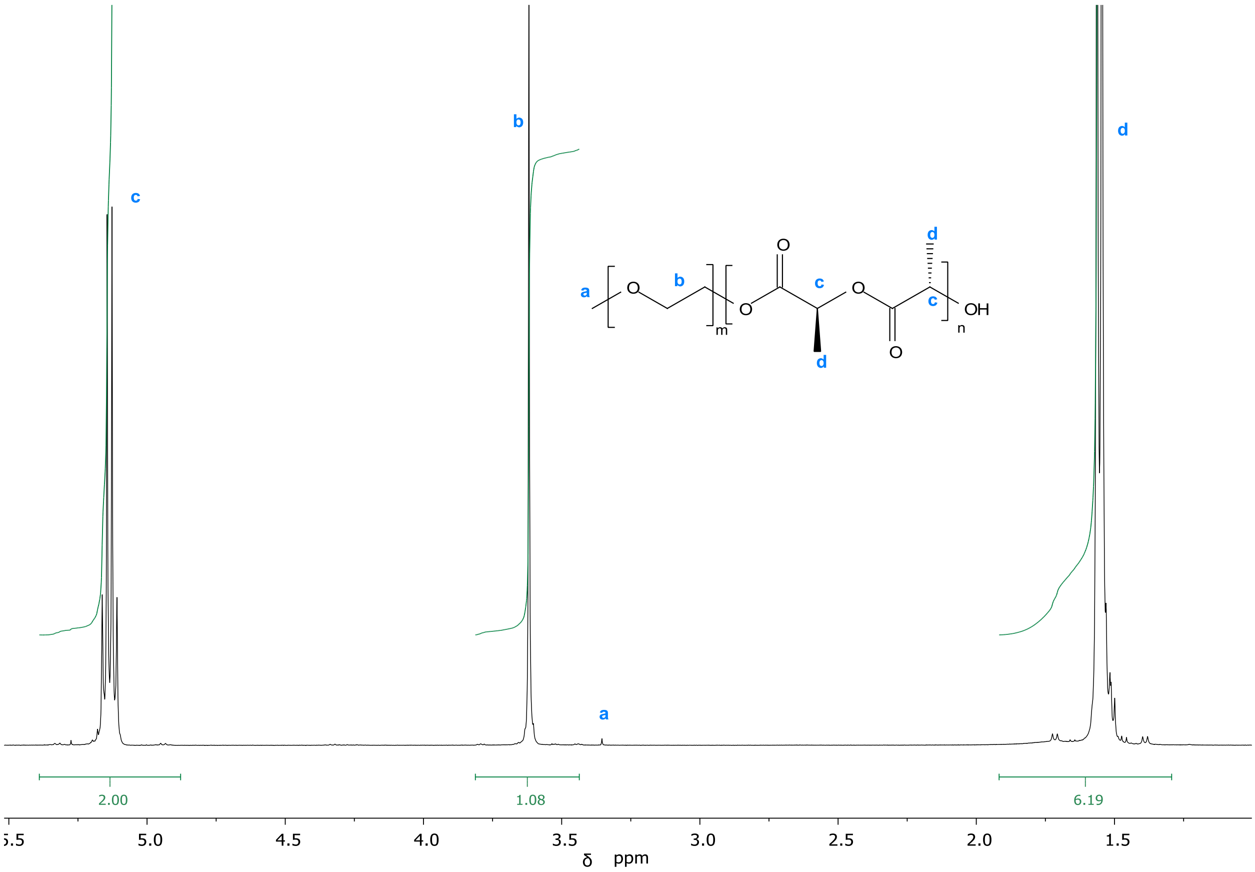

Figure S9: ^{1}H NMR spectrum of mPEG$_{5000}$—b—PLA copolymer **PLA6**.

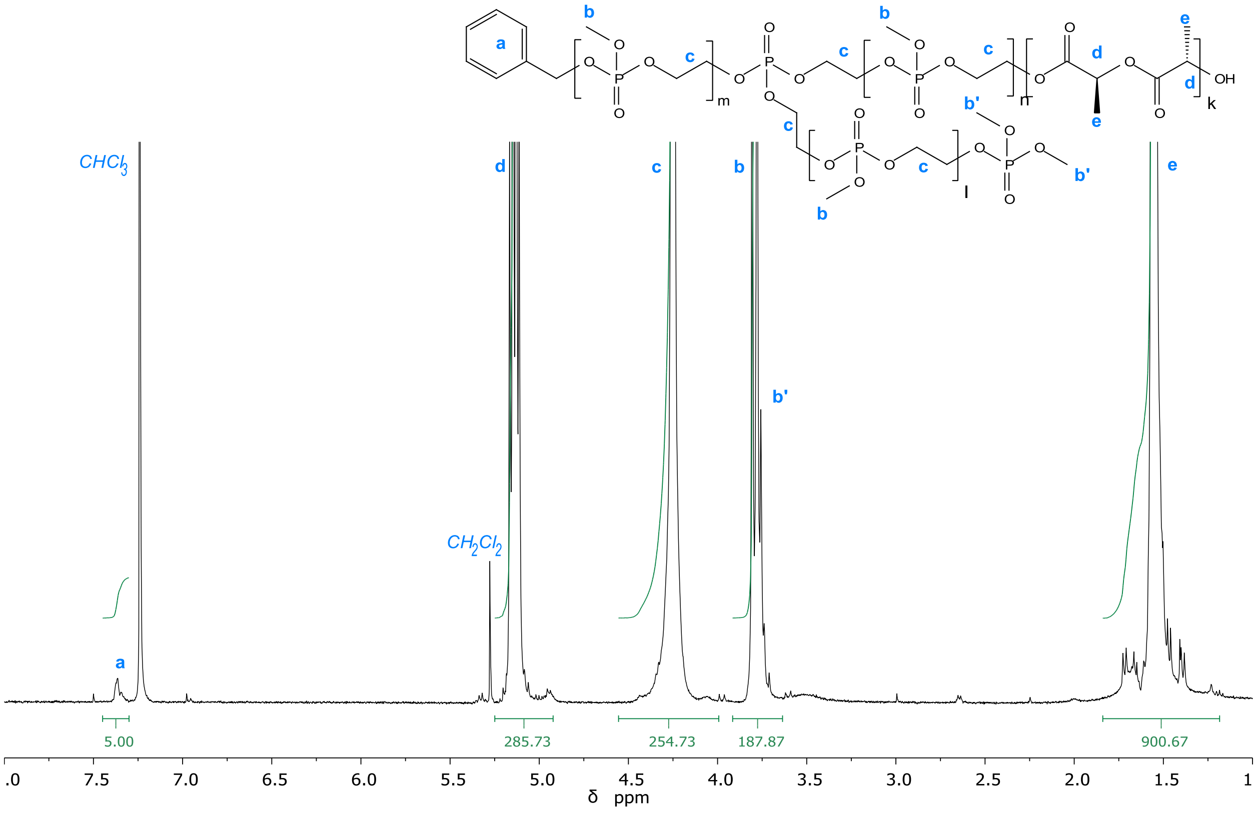

Figure S10: ^{1}H NMR spectrum of copolymer **31% PMeOEP**.

S2. Calculations

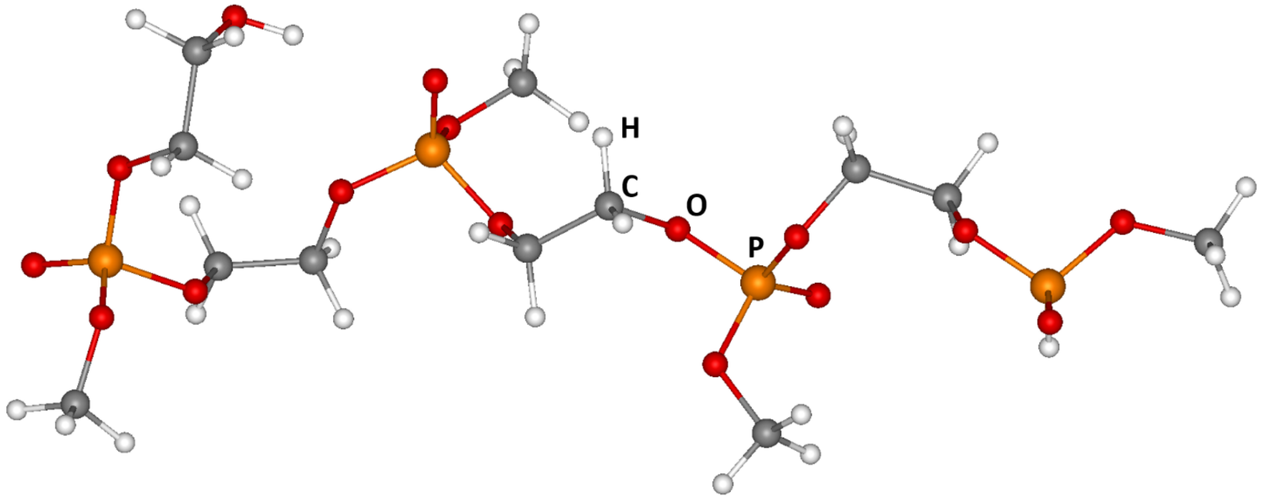

Figure S11: Calculated optimized structure of the MeOEP tetramer.

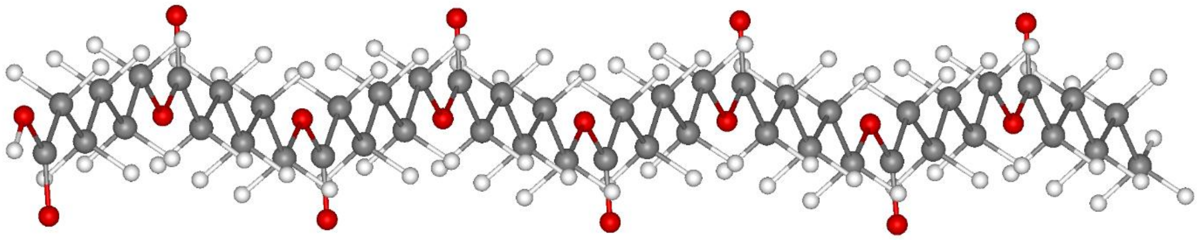

Figure S12: Calculated optimized structure of the CL octamer. Coloring of the atoms is the same as in Figure S11.

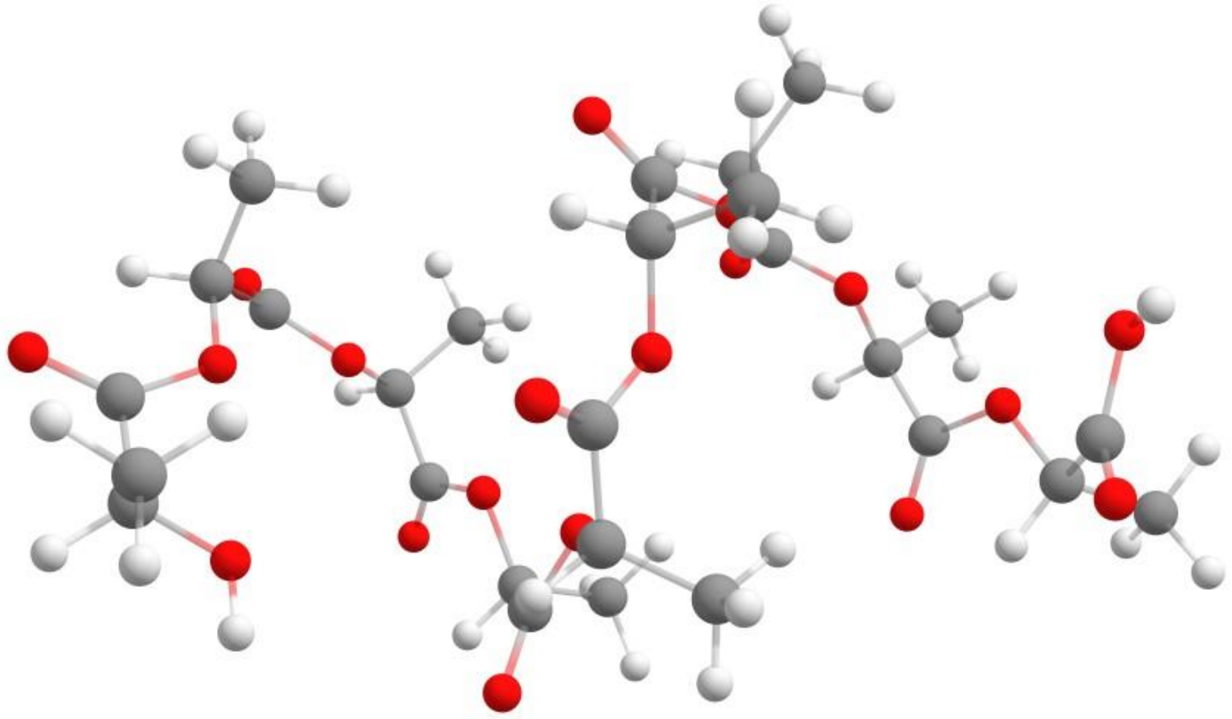

Figure S13: Calculated optimized structure of the LA nonamer. Coloring of the atoms is the same as in Figure S11.

Table S1: Harmonic frequencies (cm^{-1}) and Raman scattering activities ($Å^4$/a.m.u.) for the MeOEP tetramer in the region 100–3600 cm^{-1}. The data are presented as calculated, that is, without rounding.

Mode	Harmonic frequencies, cm^{-1}	Raman scattering activities, $Å^4$/a.m.u.
21	100.26	0.246
22	101.70	0.058
23	109.87	0.645
24	112.77	0.050
25	115.17	0.104
26	118.61	0.171
27	133.07	0.301
28	143.96	1.131
29	153.65	0.168
30	159.91	0.783
31	164.17	0.320
32	170.34	0.215
33	177.70	0.210
34	196.89	1.750
35	208.82	1.095
36	215.35	0.897
37	218.28	0.339
38	252.27	0.568
39	269.25	0.799
40	273.31	2.367
41	281.90	1.392

42	314.56	0.484
43	323.23	2.214
44	330.36	0.686
45	339.79	0.405
46	342.63	0.620
47	360.09	2.123
48	372.87	1.353
49	389.94	2.165
50	409.70	1.697
51	420.08	1.273
52	429.59	1.594
53	435.89	1.965
54	444.52	1.295
55	463.56	1.465
56	466.32	1.299
57	470.67	1.625
58	473.30	1.055
59	483.97	1.829
60	490.66	2.699
61	510.68	0.724
62	522.07	1.479
63	539.81	1.231
64	543.21	0.770
65	672.38	2.091
66	682.39	68.829
67	702.17	14.294
68	734.81	30.303
69	762.44	2.412
70	778.93	4.859
71	779.76	4.212
72	783.91	0.789
73	795.09	2.808
74	797.41	1.152
75	800.96	2.420
76	865.64	2.900
77	905.86	1.854
78	912.12	0.974
79	923.75	0.893
80	940.59	0.849
81	944.95	1.121
82	946.51	2.727
83	952.56	2.514
84	958.25	1.241
85	967.74	4.226
86	1015.67	3.367
87	1021.74	4.172
88	1026.27	6.118

89	1034.05	1.295
90	1041.71	3.674
91	1047.59	5.087
92	1054.48	1.923
93	1056.22	1.095
94	1066.76	0.083
95	1073.23	2.189
96	1077.82	1.231
97	1086.69	2.962
98	1091.12	1.568
99	1101.99	0.967
100	1105.01	2.111
101	1107.16	2.495
102	1151.94	0.662
103	1152.56	0.614
104	1152.68	0.668
105	1152.73	0.799
106	1171.24	0.887
107	1174.17	0.784
108	1174.35	0.738
109	1174.54	1.546
110	1208.37	2.945
111	1240.87	4.069
112	1246.19	8.360
113	1248.44	6.109
114	1262.92	3.754
115	1267.20	13.529
116	1268.43	4.944
117	1269.32	17.002
118	1277.28	4.373
119	1280.16	15.301
120	1295.84	6.754
121	1297.04	9.766
122	1366.52	0.180
123	1368.54	0.476
124	1368.99	0.249
125	1370.25	0.247
126	1381.12	1.871
127	1389.56	1.098
128	1391.57	1.739
129	1394.15	0.930
130	1400.61	3.688
131	1440.17	1.510
132	1440.31	2.684
133	1440.77	0.691
134	1441.44	1.263
135	1447.43	8.548

136	1451.54	5.277
137	1452.07	5.340
138	1458.56	4.326
139	1459.33	5.829
140	1459.59	5.125
141	1459.97	5.835
142	1460.76	5.812
143	1460.79	10.556
144	1462.81	2.792
145	1463.47	5.190
146	1464.60	5.395
147	1465.59	5.151
148	1467.53	5.795
149	1467.57	4.701
150	1468.83	6.465
151	2350.93	40.042
152	2928.71	132.032
153	2984.27	166.076
154	2989.25	151.069
155	2992.21	64.553
156	2993.13	157.262
157	2994.55	29.789
158	2996.43	131.809
159	2997.31	148.815
160	3002.53	244.427
161	3007.76	73.744
162	3008.45	151.811
163	3019.09	209.941
164	3031.90	140.184
165	3048.35	69.907
166	3048.43	44.096
167	3056.84	17.004
168	3061.06	19.550
169	3065.89	64.629
170	3066.77	55.228
171	3067.80	32.040
172	3068.39	54.049
173	3078.44	39.722
174	3079.48	39.129
175	3081.71	8.726
176	3092.84	76.479
177	3097.62	61.888
178	3097.70	80.183
179	3100.48	83.390

Table S2: Harmonic frequencies (cm^{-1}) and Raman scattering activities ($Å^4$/a.m.u.) for the CL octamer in the region 100–3600 cm^{-1}. The data are presented as calculated, that is, without rounding.

Mode	Harmonic frequencies, cm^{-1}	Raman scattering activities, $Å^4$/a.m.u.
44	105.05	0.094
45	106.68	0.014
46	110.02	0.003
47	112.03	0.019
48	113.97	0.019
49	117.41	0.017
50	118.09	1.577
51	119.75	0.020
52	120.98	0.014
53	121.47	0.018
54	126.58	0.007
55	128.19	0.011
56	128.94	0.141
57	129.61	0.003
58	130.96	0.007
59	133.00	0.015
60	135.31	0.001
61	137.16	0.037
62	138.38	0.060
63	143.60	0.016
64	153.26	0.003
65	158.18	0.030
66	183.63	0.795
67	185.72	0.112
68	187.04	0.019
69	187.63	0.001
70	188.49	0.005
71	189.47	0.002
72	190.28	0.010
73	190.98	0.001
74	191.45	0.059
75	196.24	0.024
76	220.06	0.787
77	223.50	0.158
78	240.86	0.024
79	242.29	0.008
80	248.23	0.610
81	254.06	0.584
82	264.38	0.129
83	275.07	0.273
84	283.60	1.170
85	296.60	1.849
86	338.01	0.062

87	347.44	0.095
88	350.75	0.654
89	352.36	0.013
90	354.97	0.007
91	355.52	0.018
92	379.72	0.186
93	387.07	0.153
94	417.86	0.570
95	420.91	0.484
96	426.49	0.066
97	432.67	0.202
98	437.26	0.128
99	439.58	0.046
100	440.30	1.921
101	501.69	0.461
102	510.97	2.051
103	517.70	0.168
104	518.16	0.707
105	518.76	0.038
106	521.37	0.029
107	525.61	0.098
108	530.21	0.011
109	533.66	5.531
110	566.92	0.225
111	567.34	0.375
112	567.41	0.098
113	567.53	0.002
114	567.67	0.191
115	567.79	0.003
116	567.91	1.076
117	618.57	4.519
118	641.31	1.310
119	693.60	3.834
120	694.78	0.517
121	696.85	0.285
122	699.33	0.087
123	701.67	1.049
124	703.53	0.017
125	704.66	13.550
126	720.68	0.067
127	725.05	0.013
128	725.07	0.007
129	725.11	0.002
130	725.15	0.002
131	725.21	0.002
132	725.24	0.012
133	725.40	0.020

134	751.91	0.032
135	754.95	0.005
136	755.44	0.002
137	756.17	0.000
138	756.98	0.005
139	757.82	0.012
140	758.50	0.004
141	758.96	0.475
142	829.13	0.153
143	829.81	0.011
144	830.87	0.007
145	832.11	0.019
146	833.39	0.005
147	834.49	0.010
148	835.26	0.052
149	839.35	0.184
150	856.51	15.029
151	879.31	1.088
152	893.21	48.146
153	896.09	0.277
154	900.32	6.215
155	905.22	0.743
156	910.01	2.889
157	913.85	1.007
158	916.52	5.406
159	949.04	0.308
160	949.46	0.039
161	949.99	19.741
162	950.10	0.719
163	950.85	0.007
164	951.60	0.006
165	951.81	0.022
166	952.24	0.003
167	952.67	0.011
168	954.26	1.391
169	956.88	0.066
170	958.62	0.213
171	959.16	0.152
172	960.70	0.173
173	961.38	0.823
174	1006.33	0.287
175	1013.74	2.107
176	1018.43	1.323
177	1019.68	0.067
178	1021.72	1.055
179	1024.14	0.636
180	1026.42	9.148

181	1027.95	67.564
182	1029.72	42.831
183	1032.45	0.309
184	1032.87	0.102
185	1033.37	0.380
186	1033.44	0.459
187	1033.77	0.002
188	1033.81	0.033
189	1051.98	5.541
190	1055.83	6.155
191	1059.14	4.308
192	1060.37	20.902
193	1060.48	2.484
194	1060.57	8.286
195	1060.60	0.257
196	1060.69	0.071
197	1060.72	0.108
198	1081.15	8.607
199	1095.28	1.058
200	1095.90	1.806
201	1096.78	0.056
202	1097.66	12.844
203	1098.31	0.008
204	1098.59	93.810
205	1104.96	0.792
206	1105.23	0.008
207	1105.61	0.106
208	1106.04	0.076
209	1106.43	0.035
210	1106.71	1.784
211	1107.66	0.884
212	1109.27	1.248
213	1109.44	10.723
214	1116.30	6.983
215	1140.27	22.350
216	1141.88	0.027
217	1143.73	0.973
218	1145.45	0.074
219	1146.84	0.385
220	1147.94	0.030
221	1148.76	0.940
222	1174.97	0.446
223	1175.05	0.023
224	1175.15	0.062
225	1175.27	0.166
226	1175.37	0.035
227	1175.44	3.612

228	1175.77	1.374
229	1203.79	1.279
230	1231.21	1.620
231	1236.36	0.221
232	1236.63	0.024
233	1237.00	0.016
234	1237.37	0.128
235	1237.67	0.002
236	1237.83	0.780
237	1241.57	4.145
238	1241.64	0.190
239	1241.75	0.318
240	1241.88	0.083
241	1242.00	0.080
242	1242.09	0.344
243	1242.44	0.559
244	1250.45	0.341
245	1270.61	1.409
246	1270.87	1.912
247	1287.06	45.780
248	1287.18	5.206
249	1287.38	2.658
250	1287.61	0.908
251	1287.84	0.869
252	1288.03	0.515
253	1288.17	1.483
254	1295.19	2.713
255	1296.49	0.007
256	1298.38	0.189
257	1300.53	0.068
258	1302.61	0.030
259	1304.14	1.574
260	1309.31	2.577
261	1309.47	0.487
262	1309.56	0.704
263	1309.68	1.748
264	1309.81	1.059
265	1309.90	4.774
266	1309.94	2.495
267	1310.31	2.289
268	1313.58	4.476
269	1314.31	14.440
270	1314.38	14.655
271	1314.43	3.228
272	1314.49	1.042
273	1314.54	0.528
274	1314.59	1.675

275	1315.31	0.516
276	1315.65	5.323
277	1316.28	1.875
278	1349.16	0.104
279	1350.40	0.012
280	1352.17	0.021
281	1354.17	0.073
282	1356.05	0.019
283	1357.44	1.254
284	1361.21	0.672
285	1363.55	0.601
286	1369.00	0.143
287	1373.69	0.654
288	1373.93	0.018
289	1374.19	0.063
290	1374.40	0.084
291	1374.53	0.226
292	1374.60	1.218
293	1377.60	0.236
294	1379.82	0.712
295	1388.82	35.663
296	1389.23	0.485
297	1389.81	3.051
298	1390.47	0.275
299	1391.12	0.818
300	1391.62	0.254
301	1391.91	2.116
302	1421.18	5.499
303	1422.04	4.986
304	1422.97	8.556
305	1422.99	0.077
306	1422.99	19.601
307	1423.01	0.102
308	1423.01	0.971
309	1423.02	1.642
310	1451.19	18.693
311	1453.80	3.534
312	1453.84	0.049
313	1453.88	1.819
314	1453.92	1.315
315	1453.96	0.285
316	1454.00	48.869
317	1454.04	66.241
318	1455.37	0.509
319	1459.13	24.802
320	1459.15	0.032
321	1459.18	0.726

322	1459.24	0.112
323	1459.30	0.048
324	1459.34	1.109
325	1459.46	3.331
326	1465.28	4.854
327	1467.90	2.284
328	1471.71	3.643
329	1471.80	0.025
330	1471.93	0.508
331	1472.05	0.053
332	1472.21	1.256
333	1472.32	0.500
334	1472.40	13.730
335	1478.60	0.576
336	1484.03	1.341
337	1484.09	0.005
338	1484.15	0.224
339	1484.22	0.016
340	1484.29	0.060
341	1484.36	0.075
342	1484.40	0.644
343	1745.65	0.236
344	1745.69	0.312
345	1745.78	0.522
346	1745.90	1.980
347	1746.04	0.041
348	1746.18	30.151
349	1746.47	15.942
350	1768.00	10.214
351	2960.29	129.047
352	2966.93	118.291
353	2967.07	97.111
354	2967.10	142.600
355	2967.11	185.422
356	2967.12	60.214
357	2967.19	85.635
358	2967.78	117.207
359	2974.13	61.425
360	2981.41	215.857
361	2986.93	140.620
362	2988.18	70.071
363	2988.45	2.714
364	2988.48	9.344
365	2988.52	35.928
366	2988.56	45.733
367	2988.59	218.149
368	2988.89	104.901

369	2989.92	253.786
370	2991.35	1130.695
371	2991.41	693.490
372	2991.47	70.810
373	2991.53	32.395
374	2991.60	16.332
375	2991.65	6.483
376	2992.12	217.968
377	2996.75	101.072
378	2996.98	87.071
379	2997.00	105.688
380	2997.02	112.524
381	2997.04	95.181
382	2997.07	101.024
383	2997.44	21.549
384	2997.88	105.851
385	2998.04	6.797
386	2998.14	8.639
387	2998.27	2.798
388	2998.44	13.689
389	2998.58	37.665
390	2998.69	182.147
391	3001.43	32.414
392	3004.61	120.490
393	3004.69	10.458
394	3004.83	9.329
395	3004.97	1.774
396	3005.14	2.300
397	3005.25	0.819
398	3005.96	14.672
399	3009.72	1.155
400	3019.64	20.501
401	3019.80	42.696
402	3019.82	10.331
403	3019.87	0.882
404	3019.91	1.149
405	3019.96	5.806
406	3020.04	69.782
407	3020.18	13.598
408	3024.82	81.801
409	3025.43	22.366
410	3025.47	4.505
411	3025.51	82.069
412	3025.54	5.633
413	3025.60	239.102
414	3025.66	155.203
415	3046.16	8.008

416	3046.55	10.958
417	3046.99	2.477
418	3047.03	0.184
419	3047.12	6.402
420	3047.18	0.410
421	3047.28	28.461
422	3047.70	10.269
423	3052.34	22.695
424	3054.02	0.421
425	3054.33	0.031
426	3054.47	0.044
427	3054.62	0.028
428	3054.81	0.256
429	3054.83	113.822
430	3055.00	0.008
431	3055.22	0.725

Table S3: Harmonic frequencies (cm^{-1}) and Raman scattering activities (Å^4/a.m.u.) for the LA nonamer in the region 100–3600 cm^{-1}. The data are presented as calculated, that is, without rounding.

Mode	Harmonic frequencies, cm^{-1}	Raman scattering activities, Å^4/a.m.u.
28	101.79	0.408
29	108.57	0.270
30	117.72	0.110
31	129.13	0.106
32	141.39	0.849
33	155.14	1.633
34	192.94	1.305
35	196.87	4.367
36	198.37	1.616
37	199.74	2.144
38	201.68	1.544
39	205.84	0.563
40	206.11	0.265
41	209.81	0.745
42	222.41	0.355
43	224.58	0.060
44	225.66	0.089
45	227.77	0.130
46	230.08	0.127
47	230.78	0.095
48	232.98	0.229
49	234.49	0.078
50	236.08	0.475
51	238.32	0.630
52	244.66	0.126

53	246.07	0.802
54	253.20	0.287
55	267.68	0.146
56	280.20	0.708
57	286.08	5.963
58	287.77	0.229
59	289.27	0.549
60	295.81	0.890
61	298.25	1.787
62	362.49	0.927
63	364.74	0.501
64	366.52	0.685
65	371.50	0.897
66	380.82	0.153
67	391.15	0.895
68	404.05	0.301
69	411.43	0.335
70	421.28	0.547
71	458.88	0.053
72	468.11	0.108
73	482.41	0.159
74	499.82	0.603
75	516.11	0.833
76	520.19	11.934
77	525.52	0.774
78	527.65	0.636
79	530.92	0.680
80	583.95	1.229
81	614.52	3.458
82	620.75	0.620
83	622.73	0.272
84	624.35	1.971
85	631.83	0.253
86	648.63	0.971
87	670.94	0.140
88	694.16	3.522
89	714.86	3.024
90	728.13	0.198
91	732.70	1.165
92	733.97	0.118
93	735.22	0.333
94	736.21	0.432
95	737.52	0.101
96	738.70	0.444
97	740.63	0.890
98	745.16	0.998
99	799.82	4.940

100	837.98	50.406
101	841.31	0.338
102	846.00	4.515
103	849.47	2.735
104	854.17	2.871
105	855.89	1.783
106	860.45	1.206
107	862.03	0.221
108	869.77	0.825
109	873.65	0.507
110	878.50	0.568
111	891.54	0.772
112	903.50	5.877
113	914.22	7.059
114	924.66	1.276
115	932.18	0.082
116	936.54	1.249
117	992.21	3.384
118	993.77	1.929
119	998.72	1.983
120	1004.50	5.455
121	1010.58	1.580
122	1015.92	0.096
123	1020.93	2.540
124	1025.25	5.204
125	1029.79	2.299
126	1033.64	4.368
127	1041.26	0.119
128	1048.84	1.280
129	1055.69	1.529
130	1057.27	2.230
131	1057.67	0.993
132	1059.01	0.663
133	1062.35	1.310
134	1068.32	4.306
135	1103.87	15.674
136	1104.38	6.517
137	1105.71	1.822
138	1108.23	2.084
139	1111.42	2.162
140	1113.56	0.736
141	1114.54	1.534
142	1116.20	0.773
143	1117.83	2.245
144	1164.70	2.407
145	1166.10	2.957
146	1230.57	6.102

147	1232.33	0.184
148	1235.54	2.482
149	1238.31	1.442
150	1238.88	1.947
151	1241.10	0.735
152	1241.35	1.839
153	1263.99	4.015
154	1302.07	1.585
155	1303.22	0.607
156	1303.81	2.824
157	1305.18	2.962
158	1305.91	9.835
159	1307.09	6.963
160	1308.55	0.637
161	1310.23	1.721
162	1315.91	3.876
163	1330.70	3.786
164	1334.29	6.947
165	1340.89	3.540
166	1342.25	1.590
167	1342.81	2.782
168	1344.40	5.469
169	1345.39	6.523
170	1345.82	2.622
171	1346.48	5.892
172	1361.48	0.319
173	1366.38	0.314
174	1366.43	0.493
175	1367.03	0.197
176	1367.70	0.190
177	1368.27	0.415
178	1368.31	0.446
179	1368.99	1.087
180	1369.41	0.970
181	1385.10	2.672
182	1455.15	5.051
183	1455.76	4.877
184	1456.17	5.069
185	1456.60	5.717
186	1456.86	3.983
187	1456.94	4.448
188	1456.98	3.141
189	1458.45	5.901
190	1458.49	4.544
191	1462.70	2.551
192	1462.83	4.165
193	1463.15	4.128

194	1463.18	2.734
195	1463.37	3.179
196	1463.52	1.710
197	1463.63	4.443
198	1464.00	4.267
199	1468.95	1.902
200	1740.98	8.888
201	1745.14	7.942
202	1747.17	6.007
203	1747.91	2.607
204	1749.17	4.573
205	1749.44	12.508
206	1750.66	18.513
207	1752.04	29.290
208	1766.82	15.369
209	2964.81	135.884
210	2996.43	183.760
211	3008.93	37.460
212	3009.33	127.138
213	3009.52	292.387
214	3009.98	139.963
215	3011.15	213.799
216	3011.53	31.400
217	3011.96	226.675
218	3012.36	243.823
219	3070.94	97.529
220	3071.13	49.666
221	3072.01	45.951
222	3073.54	64.237
223	3074.43	68.703
224	3075.31	74.599
225	3075.45	49.108
226	3076.23	79.022
227	3078.68	78.003
228	3087.76	34.529
229	3087.89	27.622
230	3088.03	106.919
231	3088.25	49.593
232	3089.72	92.248
233	3089.82	35.076
234	3090.43	58.690
235	3093.74	39.389
236	3094.79	68.288
237	3100.45	39.159
238	3100.62	40.776
239	3103.49	33.366
240	3104.93	43.297

241	3106.01	36.364
242	3106.28	29.486
243	3106.95	50.626
244	3109.89	39.634

Table S4: Four-exponential (4z) basis set contraction patterns.

Atom	Orbital basis	
	**Primitive**	**Contracted**
H	(8s3p2d)	[4s3p2d]
C	(14s8p3d2f)	[8s4p3d2f]
O	(14s8p3d2f)	[8s4p3d2f]
P	(20s15p3d2f)	[13s9p3d2f]
